# Social Skills and Peer Relationships as Serial Mediators Between Mindfulness and Spiritual Well-Being in Adolescence

**DOI:** 10.3390/healthcare14010054

**Published:** 2025-12-25

**Authors:** Mehmet Akif Kay, Ümit Kahraman, Betül Kapkın İçen, Amine Nur Arıkan, Osman Tayyar Çelik, Mehmet Emin Çay

**Affiliations:** 1 Department of Child Care and Youth Services, Vocational School of Social Sciences, Batman University, 72100 Batman, Türkiye; mehmetakif.kay@batman.edu.tr; 2 Department of Child Development, Faculty of Health Sciences, Bilecik Şeyh Edebali University, 11100 Bilecik, Türkiye; umit.kahraman@bilecik.edu.tr; 3 Child Development Program, Department of Child Care and Youth Services, Vocational School of Health Services, Inonu University, 44210 Malatya, Türkiye; 4 Department of Child Development, Faculty of Health Sciences, Inonu University, 44210 Malatya, Türkiye; nur.arikan@inonu.edu.tr (A.N.A.); otayyar.celik@inonu.edu.tr (O.T.Ç.); mehmet.cay@inonu.edu.tr (M.E.Ç.)

**Keywords:** mindfulness, spiritual well-being, social skills, peer relationships, adolescence

## Abstract

**Objectives**: This study aimed to investigate the mediating roles of social skills and peer relationships in the association between mindfulness and spiritual well-being (SWB) among adolescents. Drawing on the mindfulness-to-meaning theory, the research sought to clarify how mindfulness supports adolescents’ spiritual well-being through social and relational mechanisms. **Method**: A correlational research design was employed with a sample of 761 adolescents attending high schools in Türkiye. Data were collected using the Mindful Attention Awareness Scale, Social Skills Scale, Peer Relations Scale, and the Three-Factor Spiritual Well-Being Scale. The hypothesized serial mediation model was tested using PROCESS Macro Model 6 with 5000 bootstrap samples. **Results**: Mindfulness was positively associated with SWB. Both social skills and peer relationships showed significant mediating effects. The serial indirect effect through social skills and peer relationships was also significant. **Conclusions**: Findings highlight mindfulness as a key psychosocial resource that enhances adolescents’ spiritual well-being through improved social skills and supportive peer relationships. School-based mindfulness programs should integrate peer interaction and social skills components to promote adolescents’ holistic development.

## 1. Introduction

Adolescence is a period marked by hormonal changes, rapid physical growth, development in mental functions, emotional changes, and social maturation. This period is also characterized as a turbulent and stressful time marked by complex social interactions and increasing academic and social pressures [1,2]. Consequently, the likelihood of various psychological and emotional problems affecting adolescent well-being increases during this period [3,4]. Indeed, it is reported that mental health problems such as depression and suicide affect approximately two out of ten adolescents worldwide and that risky health behaviors reach alarming levels during this period [5]. Furthermore, the effects of mental health outcomes during adolescence extend into adulthood [6]. Parallel to the paradigm shift in psychology, there is an increasing emphasis on promoting positive characteristics such as life satisfaction and well-being as preventive mechanisms rather than focusing on pathology in adolescents [7,8]. In this context, SWB is considered a critical factor in promoting adolescent well-being and preventing psychological health impairments [6,9].

Research indicates that adolescents with higher levels of SWB tend to have greater life satisfaction [10], experience lower levels of psychological distress [11], and exhibit lower tendencies toward risky behaviors such as gaming addiction [12]. While research results generally suggest that SWB has potential value in promoting adolescent well-being, research on how to promote SWB in adolescents is still in its infancy. However, countries that fail to develop the necessary intervention programs and policies to support adolescent well-being risk compromising their healthy population structure, including their socioeconomic development [13].

While previous empirical studies have provided evidence on the link between SWB and mindfulness [14,15], propositions from the mindfulness to meaning theory [16] provide support for the connections between the variables. However, existing research still falls short in explaining how mindfulness promotes SWB in adolescent populations. Building on this gap, this study positions social skills and peer relationships—critical components of adolescence—as potentially interrelated mediating mechanisms in the relationship between mindfulness and SWB. We propose a model for these relationships between variables and aim to test this model with real data. From a public health perspective, adolescence is a sensitive developmental period in which risk and protective factors are consolidated and shape long-term health outcomes. Therefore, identifying modifiable psychosocial processes associated with SWB can inform low-intensity preventive approaches that can be implemented in schools and community settings.

### 1.1. Literature and Research Hypotheses

Discussions about mindfulness and its applications are based on Buddhist teachings and are addressed in three fundamental dimensions. These are: momentary awareness, ethics, memory, and recollection. The inclusion of mindfulness in scientific research has been made possible through approaches and definitions that integrate mindfulness into the field of psychology [17]. In line with psychology, mindfulness is defined as purposeful and conscious focus on what is happening in the moment without judgment, reaction, or analysis [18]. Brown and Ryan [19] define mindfulness as the process of observing and experiencing current experiences with high sensitivity and attention, without criticism. These definitions imply that mindfulness is a conscious action. Furthermore, the common point among these definitions is that mindfulness is a way of directing attention [20].

Research on mindfulness, along with conceptualizations appropriate to the science of psychology, has been addressed in a wide range of both clinical and non-clinical settings [21]. Mindfulness is being addressed through different research approaches and is increasingly associated with positive outcomes and indicators of well-being [22]. Recent research on mindfulness in adolescents has shown promising results in improving mental health outcomes and supporting well-being [1]. For example, an experimental study by Scafuto et al. [23] aimed to reduce internalization problems experienced by children and adolescents at school and promote their well-being. The study reported that a mindfulness program was effective in increasing personal growth and life purpose, which are components of psychological well-being. Vieira and Faria [4] revealed a positive relationship between mindfulness and school success and emotional intelligence in their cross-sectional studies, while a longitudinal study conducted by Sheng, Liu, Wang, Yu and Xu [8] determined that mindfulness affects subjective well-being in adolescents through the chain mediation effect of self-esteem and rejection sensitivity. Furthermore, a meta-analysis examining the effectiveness of mindfulness interventions found that mindfulness activities have significant effects on mental health and well-being outcomes, particularly in late adolescence [24]. Although studies consistently reveal these positive effects of mindfulness, its relationship with SWB, which is defined by the WHO as the fourth dimension of health [25], has been little explored.

### 1.2. Spiritual Well-Being

Spirituality is defined as internal experiences related to transcendence and belief in invisible phenomena [26]. Although this definition associates spirituality more with religiosity, spirituality is not solely a component of religion. Spirituality encompasses broader terms that also frame religiosity [27,28]. In this context, spirituality is concerned with the search for meaning and value in a person’s interaction with themselves and their environment [12]. This interaction refers to the relationship with God or a transcendent being in the vertical dimension and the interaction with oneself, others, and the environment in the horizontal dimension [29]. A more comprehensive definition of spirituality, linking it to well-being, was provided by Fisher [30]. Accordingly, spirituality or SWB is the state of being perceived as being in harmony with oneself, other individuals (society), the environment (nature, etc.), and the transcendent [31]. In addition, the WHO’s definition of mental health as part of overall health has sparked widespread SWB research in the fields of psychology and health [25].

Numerous studies conducted with adolescents [6,32,33] have shown that SWB is associated with life satisfaction, psychological well-being, anxiety, depression, suicidal ideation, happiness, and psychological resilience. A systematic review of 241 studies by Hardy et al. [34] emphasized that spirituality is a protective factor against risky behaviors such as addiction and health problems. Additionally, research by Benson et al. [35] with a large sample of adolescents from different countries proposed two fundamental mechanisms for spiritual development in adolescents. The first is the understanding of God or a transcendent power, which is related to spiritual and religious practices. The second refers to psychological processes involving awareness, the search for meaning, and connection through social beliefs. As evidence accumulates that SWB increases adolescent well-being and acts as a protective factor, the field of research is likely to expand further. Additionally, the beginning of research on the neurodevelopmental foundations of spirituality [36] encourages interdisciplinary studies and broadens the field. Finally, De Souza [37] emphasizes that adolescents, surrounded by very different lifestyles, role models, and powerful media, are trapped in a materialistic world and that adolescents who lack a solid spirituality based on family and community relationships will experience alienation, highlighting the need to focus on factors that promote SWB.

### 1.3. The Relationship Between Mindfulness and SWB

The mindfulness to meaning theory and empirical research results provide convincing evidence that mindfulness can support SWB. This theory [16], which emphasizes how meaning is constructed through mindfulness, suggests that mindfulness will increase reappraisal, enable a transition to a metacognitive state, and thereby nurture positive emotions and a sense of meaning. Furthermore, the mindfulness to meaning theory predicts that mindfulness will enable the reappraisal of life circumstances and one’s abilities and values, providing opportunities for growth and transformation [8,38,39]. Therefore, mindfulness provides individuals with the opportunity for spiritual development through accepting their flaws and self-evaluation [40].

There is a range of research providing strong evidence for the relationship between mindfulness and SWB. This line of research addresses mindfulness in clinical and non-clinical, adolescent and adult contexts. A study with individuals undergoing substance use treatment [41] found that higher mindfulness was associated with increased spirituality, while in visually impaired individuals, mindfulness promoted SWB through interpersonal connection [42], and a study on gifted high school students [43] found that mindfulness significantly predicted SWB in a positive direction. Additionally, experimental evidence supporting that mindfulness-based interventions increase SWB is increasingly robust [44,45,46]. In conclusion, both theoretical and empirical evidence indicates that mindfulness serves as a supportive structure for SWB. Within this context, the first hypothesis of the study is formulated as follows:

**H1.**  *Mindfulness in adolescents is positively related to SWB.*

### 1.4. Social Competence and Peer Relationships as Serial Mediators

Social skills are a goal-oriented process that begins with the accurate perception of a social situation, followed by the ability to behave appropriately according to the social situation, and finally, the ability to adjust one’s own behavior according to changing social situations. Social skills are widely regarded as among the most essential life skills for individuals’ social well-being [47]. They play a pivotal role in the healthy development and maintenance of peer relationships [48]. Within the social competence approach, Cavell [49] conceptualizes social skills as a repertoire of behaviors that enables children to engage in effective, reciprocal, and goal-directed interactions with peers. Competencies such as communication, cooperation, empathy, and problem solving help children elicit positive peer feedback and sustain peer relationships. Conversely, deficiencies in social skills are closely linked to peer rejection and social isolation [50]. Consistent with this view, children with richer social and emotional skill repertoires tend to form higher-quality peer relationships [51] and are more likely to initiate and maintain peer interactions than their less skilled counterparts [52]. Peer relationships among adolescents are a relational domain within social environments that should be high-quality, consistent, and reciprocal [53]. In parallel, the beneficial influence of mindfulness on interpersonal relationships has been theoretically grounded [54]. Shapiro et al. [55] propose that mindfulness enhances self-regulation through processes of reappraisal shaped by intention, attention, and attitude. This, in turn, supports individuals’ capacity to respond to their environment in more adaptive and health-promoting ways.

Adolescents’ social skills constitute a multifaceted construct encompassing emotion-al, social, and psychological dimensions, with empathy, tolerance, and respect as core components [56]. Mindfulness may enhance these skills by fostering interest and empathy, facilitating coping with difficult emotions, and strengthening social connectedness through perspective-taking and reduced prejudice [54,57]. In turn, stronger social skills are likely to promote more positive peer relationships [9]. From a conceptual and develop-mental standpoint, social skills are a fundamental prerequisite for establishing and maintaining peer relationships [51]. Consistent with this view, the Social and Emotional Learning approach highlights competencies related to recognizing, expressing, and regulating emotions, as well as understanding others’ emotions and responding appropriately within developmental contexts [58]. Adolescents who experience positive and supportive peer relationships show better social-emotional adjustment, higher self-efficacy, and greater social competence [59], and such relationships are also associated with enhanced spiritual well-being alongside broader mental and physical health [60,61]. Accordingly, mindfulness may contribute to adolescents’ spiritual well-being indirectly by strengthening social skills and fostering supportive peer relationships [26,62,63]. Based on these theoretical considerations and empirical findings, we formulated the following hypotheses regarding the mediating roles of social skills and peer relationships:

**H2.**  *Social skills play a mediating role in the relationship between mindfulness and SWB in adolescents.*

**H3.**  *Peer relationships play a mediating role in the relationship between mindfulness and spiritual well-being in adolescents.*

**H4.**  *Social skills and peer relationships play a serial mediating role in the relationship between mindfulness and spiritual well-being in adolescents.*

### 1.5. Present Study

Previous literature has well documented the relationship between mindfulness and adolescent well-being indicators [2,54]. However, the mechanisms explaining the relationship between mindfulness and SWB, which is also considered an important dimension of health and a well-being indicator, remain unclear in adolescents. The theory of meaning from mindfulness [16] and empirical research findings suggest that social competence and peer relationships may be important mechanisms explaining the relationship between these variables. This study explains internal, behavioral, and interpersonal variables within a single model to assess adolescents’ SWB more holistically. Furthermore, the study deepens existing empirical evidence by using a sample from outside Western culture. In this context, the study aims to explain how the connection between mindfulness and SWB is shaped through social skills and peer relationships, taking into account the social and emotional developmental dynamics specific to adolescence. The results obtained in this context will contribute theoretically to the field of developmental psychology and lay the groundwork for the development of comprehensive intervention programs to support adolescents’ psychological and mental well-being.

## 2. Materials and Methods

### 2.1. Research Model

**Figure 1:**
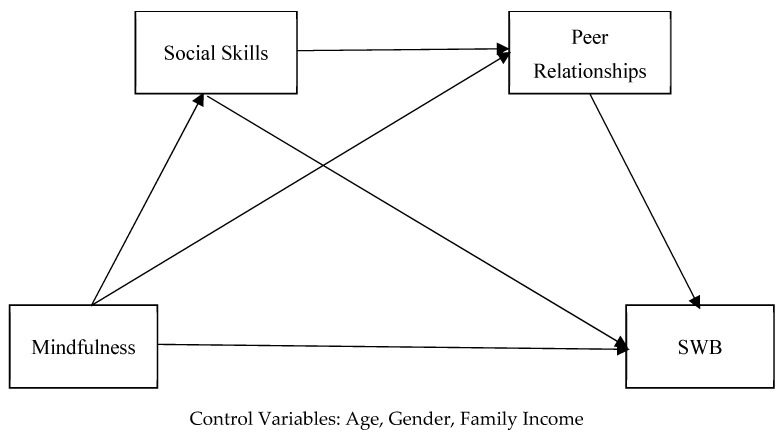
Research model.

A serial multiple mediation analysis was conducted to examine the mediating roles of social skills and peer relationships in the relationship between mindfulness and SWB in adolescents. Serial multiple mediation analysis is a type of modeling that involves two or more causally related mediating variables [64]. The model illustrating the mediating roles of social skills and peer relationships in the relationship between adolescents’ mindfulness and SWB is presented in Figure 1.

As shown in Figure 1, the effect of mindfulness on SWB has been modeled through four different pathways, and this model includes two mediating variables. The first indirect effect reaches SWB through social skills, the second indirect effect through peer relationships, and the third indirect effect through both social skills and peer relationships. The fourth pathway represents the direct effect of mindfulness on SWB. In order to increase the reliability of the research results, the variables of gender, age and family income level were included in the serial mediation analysis as control variables (covariates).

### 2.2. Participants and Procedure

The participants in the study consisted of 761 high school students attending schools in Malatya, Türkiye. Of the participants, 356 (46.7%) were female and 405 (53.3%) were male. Students were enrolled in four different grade levels: 9th grade (n = 124, 16.3%), 10th grade (n = 211, 27.7%), 11th grade (n = 245, 32.2%), and 12th grade (n = 181, 23.8%). Participants’ ages ranged from 13 to 18, with a mean age of 15.22 (SD = 0.97). Regarding socioeconomic status, 187 (24.6%) adolescents were from low-income families, 412 (54.1%) from middle-income families, and 162 (21.3%) from high-income families.

Research data were collected by researchers from 13 different secondary schools affiliated with the Ministry of National Education in Malatya province. To ensure the representativeness of the sample, schools were selected using stratified sampling based on school type, representing different academic achievement levels. Following the necessary approvals from the institutional review board and ethics committee, an informed consent procedure was implemented. Consent forms explaining the purpose of the study and the principles of confidentiality were sent to the students’ parents or legal guardians. Only adolescents whose parents gave written permission and who themselves verbally agreed to participate were included in the study. The data collection process was carried out between 1 May and 5 June 2025, during which questionnaire forms were distributed to volunteer students during school visits. In this context, data was collected from 792 adolescents, but the responses of 31 participants who filled out the questionnaire form incompletely and randomly were excluded, and thus the analyses were performed on 761 data points. To evaluate the adequacy of the sample size (N = 761), a post hoc power analysis was conducted using G*Power (v3.1). Based on a model including six predictors (the independent variable, two mediators, and three control variables) and an alpha level of 0.05, the analyses were estimated to have a statistical power of 0.84 to detect even small effect sizes (Cohen’s f^2^ = 0.02). This value exceeds the commonly accepted threshold of 0.80 [65], indicating that the sample size was statistically adequate.

### 2.3. Measurements

#### 2.3.1. The Mindful Attention Awareness Scale

The Mindful Attention Awareness Scale developed by Brown and Ryan [19] was adapted into Turkish by Özyeşil et al. [66]. The scale measures awareness of momentary experiences in daily life and the general tendency to be mindful of these situations. It consists of 15 items and a single dimension. The scale is rated on a 6-point Likert scale (“almost always = 6”, “almost never = 1”). Sample items are as follows: “I break or spill things because of carelessness, not paying attention, or thinking of something else.”, “I forget a person’s name almost as soon as I’ve been told it for the first time.” High scores on the scale indicate high mindfulness. The Cronbach’s Alpha value of the original scale is 0.82, and that of the Turkish version is 0.80.

#### 2.3.2. Social Skill Scale

The Scale developed by Kocayörük [67] measures social skills such as making eye contact, listening, initiating conversation, maintaining conversation, and engaging in conversations appropriate to daily life situations. The scale consists of 20 items and a single dimension, rated on a 4-point Likert scale (“completely = 4”, “not at all = 1”). Sample items on the scale are as follows: “I speak comfortably in a group,” “I make amends with people I have hurt or upset.” A high score on the scale indicates competence in demonstrating higher social skill behaviors. In the original version of the scale, the authors calculated Cronbach Alpha value as 0.75.

#### 2.3.3. Peer Relationship Scale

Developed by Kaner [68] to assess adolescents’ relationships with their peers. The Peer Relationships Scale consists of 4 subscales: “affection,” “trust and identification,” “self-disclosure,” and “loyalty,” and a total of 18 items. The scale is rated on a 5-point Likert scale (“always = 5,” “never = 1”). Sample items on the scale are: “My friends like me,” “My friends care about my problems,” and “When I have problems, my friends help me.” A high score on the scale indicates positive peer relationships. The Cronbach alpha value in the original scale was calculated as 0.86.

#### 2.3.4. The Spiritual Well-Being Scale

The scale developed by Ekşi and Kardaş [69] consists of three subscales and 29 items. The subscales of the scale are transcendence, harmony with nature, and anomie. The scale is rated on a 5-point Likert scale (“completely agree with me = 5”, “completely disagree with me = 1”). Sample items are as follows: “Belonging to a divine power gives me confidence,” “I treat all living things on earth well,” “I have not yet found the purpose of my life.” Items in the scale’s anomie subdimension are reverse-scored. High scores on the scale indicate an increase in spiritual well-being. The Cronbach alpha value for the original scale was calculated as 0.86.

### 2.4. Data Analysis

The study first examined the presence of common method bias, conducted reliability analyses, and tested the measurement model. Subsequently, the normality assumption was examined, and descriptive statistical results were reviewed. Correlation analyses were performed to determine the relationship between variables. PROCESS Macro Model 6 was used to determine the serial mediating roles of social skills and peer relationships in the relationship between adolescents’ awareness and spiritual well-being.

## 3. Results

### 3.1. Measurement Model and Preliminary Analyses

Prior to testing the mediation model, preliminary analyses were conducted to assess common method bias and test the validity and reliability of the measurement model.

#### 3.1.1. Common Method Bias

Since the data in our study were collected from a single source and were self-reported, we attempted to control for common method bias by applying several procedural remedies in line with recommendations accepted in the literature [70,71]. During the construction of the questionnaire, measurement tools with clear, concise, and unambiguous statements were used to ensure participants understood the questions correctly. Furthermore, participants were encouraged to provide honest answers by being assured that their responses would remain anonymous and that there were no right or wrong answers. To prevent participants from inferring relationships between variables and producing artificially consistent responses, the titles of the scales were not included in the questionnaire [70].

In addition to these procedural measures, we also statistically tested for the presence of CMB using Harman’s single-factor test [72]. As a result of the exploratory factor analysis, 16 factors with eigenvalues above 1 were identified. The highest eigenvalue obtained was 16.767%, indicating that the majority of the data was not explained by a single factor. This can be interpreted as suggesting that there is no significant problem in terms of common method bias.

#### 3.1.2. The Measurement Model

A CFA including all variables was conducted to test the measurement model. The fit indices obtained (RMSEA = 0.06; CFI = 0.94; TLI = 0.92) indicate that the measurement model fits well. The Average Variance Extracted (AVE) values, one of the most commonly used criteria in assessing convergent validity, were examined. AVE values above the accepted threshold of 0.50 (see Table 1) indicate that convergent validity is achieved in the model [73].

Additionally, a divergent validity analysis was conducted to determine whether the scale structures were distinct from one another. In this context, the root mean square error of approximation (RMSEA) value proposed by Fornell and Larcker [74] and the Heterotrait-Monotrait ratio (HTMT) value proposed by Henseler et al. [75] were examined. According to the Fornell and Larcker [74] criterion, the square root of each scale’s AVE value should be greater than its correlation value with other scales. The AVE root values in Table 2 are greater than the correlation values. Furthermore, HTMT values below 0.90 indicate that there is sufficient discriminant validity between the structures. When all the results obtained are evaluated together, it can be said that the measurement model provides construct validity.

### 3.2. Descriptives and Correlations

We begin by presenting our findings, starting with descriptive statistics and correlation analysis results. These analysis results are presented in Table 2. Accordingly, mindfulness is positively correlated with social skills (r = 0.272, *p* < 0.001), peer relationships (r = 0.348, *p* < 0.001), and SWB (r = 0.346, *p* < 0.001) in adolescents. Similarly, social skills are positively related to peer relationships (r = 0.435, *p* < 0.001) and SWB (r = 0.571, *p* < 0.001). Finally, peer relationships are also positively related to SWB (r = 0.432, *p* < 0.001).

### 3.3. Test of Mediation Effects

**Figure 2:**
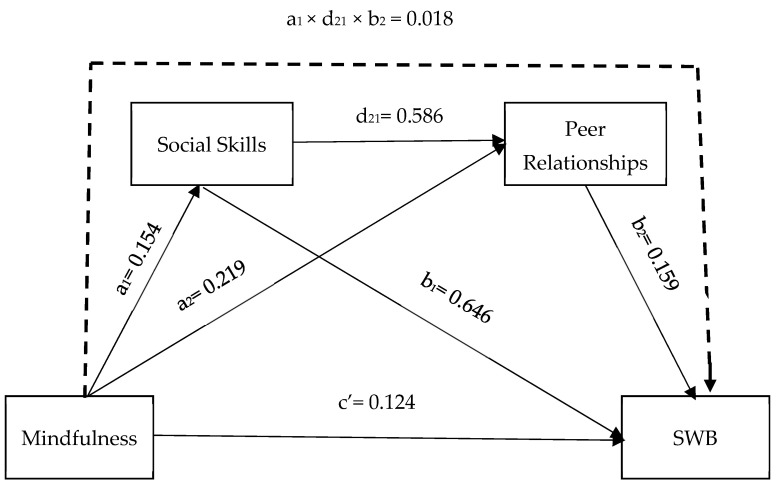
Direct and indirect effects. Note. a_1_ × d_21_ × b_2_ indirect effect.

Statistical results regarding regression coefficients and mediating effects are presented in detail in Table 3 and Figure 2, along with the modeled pathways. According to the information in Table 3, mindfulness is a significant predictor of social skills (β = 0.154; *p* < 0.01), peer relationships (β = 0.219; *p* < 0.01), and spiritual well-being (β = 0.124; *p* < 0.01) and is positively related to them.

To test the mediating role of social skills and peer relationships between mindfulness and spiritual well-being, an analysis was conducted using the Bootstrap method (5000 resamples) with a 95% confidence interval. The analysis findings revealed that social skills and peer relationships played a significant mediating role in this relationship. Mindfulness indirectly affects SWB through social skills (β = 0.099; CI = 0.064–0.138) and peer relationships (β = 0.035; CI = 0.019–0.052). Additionally, there is a serial mediating effect of social skills and peer relationships (β = 0.018; CI = 0.009–0.029) in the relationship between Mindfulness and SWB. This effect is statistically significant because the calculated confidence interval does not include zero, indicating that the indirect effect is meaningful [64]. The total indirect effect in the model was calculated as 0.149; the total effect was calculated as 0.273. The research results reveal that adolescents’ high levels of mindfulness increase SWB and that this effect is significantly strengthened through social skills and peer relationships.

## 4. Discussion

This study examined the relationship between mindfulness and SWB among adolescents in a Turkish sample and the mediating roles of social skills and self-esteem in this relationship. Our results showed that mindfulness in adolescents has a direct and positive relationship with SWB. Furthermore, our analysis results confirmed the sequential mediating effects of social skills and peer relationships in the relationship between mindfulness and SWB in adolescents.

Our first hypothesis proposed that mindfulness in adolescents is positively related to SWB. Our analyses confirmed this hypothesis and showed that adolescents with mindfulness tend to have higher SWB. In other words, the results indicate that adolescents’ mindfulness supports SWB. This result is consistent with previous research results [43,44,76]. Mindfulness can encourage deeper connections with both the individual and their environment by promoting self-awareness [77]. This suggests that mindfulness can make a complementary contribution to spiritual life [15]. Our results also support the fundamental assumptions of the theory of meaning from mindfulness. Our findings empirically validate the ‘positive reappraisal’ mechanism of the mindfulness-to-meaning theory within the context of adolescent spirituality. Keeping these explanations in mind, it can be said that mental mindfulness can be a driving force for increasing spiritual well-being in adolescents. However, it should be noted that this relationship is shaped by cultural and religious norms in the context of Turkish adolescents. In Turkey, the concept of spirituality is mostly perceived in conjunction with religious moral values [78]. Children’s emotional regulation skills depend not only on their development and age but also on the environment in which they grow up [79]. Cross-cultural comparisons reveal that parenting practices and emotional socialization processes vary according to culture. This indirectly affects children’s awareness and emotional regulation experiences [80].

The second hypothesis of our study proposed that social skills play a mediating role in the relationship between mindfulness and SWB in adolescents. Our results showed that mindfulness in adolescents has an indirect relationship with SWB through social competence. In other words, mindfulness in adolescents promotes better social skills, which in turn positively affects SWB in adolescents. There is well-developed literature on mindfulness positively affecting the core components of social skills. For example, Schonert-Reichl and Lawlor [20] found that mindfulness activities improved social and emotional competence in adolescents, while Jones and Hansen [57] examined the relationship between mindfulness and supportive communication in three exploratory studies, determining that mindfulness affects supportive communication by developing communicative coping, reappraisal, and social skills. In addition, it can be said that individuals with better social skills will have more positive evaluations related to themselves in the context of SWB [29]. Indeed, social skills have been associated with well-being and more positive emotions in adolescents [2,81]. Although there are no studies that simultaneously examine the relationship between mindfulness, social skills, and spiritual well-being in adolescents, considering the above-mentioned results and theoretical explanations, it can be said that social skills in adolescents act as a functional bridge between mindfulness and SWB.

Another hypothesis of our study was that peer relationships play a mediating role in the relationship between mindfulness and SWB in adolescents. Our analyses revealed that higher mindfulness also affects SWB by promoting more positive peer relationships. These findings are consistent with the results of previous studies examining the relationship between peer relationships and spiritual well-being. Current studies emphasize the importance of peer support in developing SWB and gaining existential meaning in children and adolescents [26,62]. Additionally, research has well-documented the connection between peer relationships and adolescents’ well-being [82,83]. In this context, quality peer relationships lead to a positive assessment of one’s relationship with oneself. Furthermore, peer relationships contribute to the deepening of interpersonal relationships in the context of spiritual development and help individuals construct their own identity and self-knowledge [26]. In light of these explanations, considering the positive effect of mindfulness on social relationships [9], we emphasize that peer relationships have good potential as a means of indirectly promoting mindfulness’s SWB. In addition to these results, our findings indicate that social skills are a stronger mediator than peer relationships. Mindfulness directly supports adolescents’ social behavior by strengthening self-regulation processes such as attention control and emotion regulation; therefore, social skills may demonstrate a stronger mediating effect as a more ‘direct’ mechanism conveying mindfulness’s impact on well-being [84,85]. In contrast, peer relationships, while drawing on the same skills, may reflect a relatively weaker mediating effect as they are more sensitive to contextual factors such as class or school [86,87].

The final hypothesis of our study was that social skills and peer relationships play sequential mediating roles in the relationship between mindfulness and SWB in adolescents. In this context, our findings confirmed the sequential mediating roles of social skills and peer relationships in the relationship between mindfulness and SWB in adolescents. More explicitly, our results show that mindfulness is not only directly related to SWB, but also enhances social skills, improves peer relationships, and indirectly supports SWB through this process. In other words, mindfulness nurtures social skills such as empathy, communication, and interpersonal problem-solving in adolescents; these skills, in turn, promote higher-quality peer relationships, thereby strengthening SWB [88,89]. This result is consistent with theoretical approaches and previous research [16,42] emphasizing that mindfulness supports social functioning and spiritual development by reinforcing meaning and purpose. In this context, the relationship between mindfulness and SWB can be said to be multidimensional and complex. However, our results provide a robust explanation by offering evidence for the serial mediating roles of social skills and peer relationships in elucidating the complex relationship between these constructs.

## 5. Theoretical and Practical Implications

From a preventive healthcare and health promotion perspective, our findings suggest that mindfulness-based programs aimed at enhancing adolescents’ SWB can be strengthened by systematically integrating social skills training and structured peer interaction components. Given the stronger mediating role of social skills, intervention protocols may benefit from explicitly targeting communication, empathy, appropriate emotional expression, conflict resolution, and cooperation through manualized, skills-based activities delivered alongside mindfulness practices. These components are consistent with upstream, low-intensity prevention approaches that can be implemented in school and community settings to bolster protective psychosocial resources before clinically significant difficulties emerge.

Moreover, the mediating role of peer relationships indicates that efforts to promote adolescents’ SWB should extend beyond individual-level skill acquisition and attend to the relational climate in which adolescents’ daily experiences are embedded. Accordingly, embedding mindfulness practices within broader school-based health promotion frameworks (e.g., social–emotional learning or positive youth development initiatives) and incorporating peer-supported formats (e.g., small-group practice, collaborative exercises, peer feedback) may increase both reach and sustainability.

From a theoretical perspective, our findings make a significant contribution to the development of the mindfulness to meaning theory by adapting the proposed mindfulness–well-being process to adolescence. The results demonstrate that the effect of mindfulness on SWB operates not only through individuals’ internal cognitive reappraisal processes but also through social and relational mechanisms. In this respect, the study suggests that the meaning-making process, which has been predominantly conceptualized at the individual level within the mindfulness-to-meaning theory, should be understood as a structure that expands within the social context during adolescence.

## 6. Limitations and Future Research

There are some limitations to consider when interpreting our research results. First, since a cross-sectional research design was adopted, the relationships between variables should not be interpreted in terms of causality. Longitudinal and experimental designs are required to determine causal relationships between these variables. Second, although we took application-based and statistical precautions, our data collection tools were based on self-report scales, so socially desirable responses may have been mixed into the answers. In the future, researchers can address this limitation by collecting data from different sources. Additionally, the sample of our research consists of adolescents in Turkish culture. It is recommended that the relationships between variables be tested in different cultures and societies. Finally, the research data was collected from adolescents attending high school in Malatya, Türkiye. In this context, more research is needed to determine the generalizability of the research results to different cultural contexts.

## 7. Conclusions

This study proposes and tests a conceptual model of the relationship between mindfulness, social skills, peer relationships, and spiritual well-being in adolescents. Our results provide important insights into the direct and indirect effects of mindfulness on SWB in adolescents. In this context, our research results emphasize the importance of mindfulness in enhancing SWB in adolescents. In addition, our results reveal that social skills and peer relationships are critical mediators in promoting spiritual well-being in adolescents.

## Figures and Tables

**Table 1:** Reliability, convergent validity, and HTMT results.

Variables	α	AVE	1	2	3	4
(1) Mindfulness	0.85	0.517	1.000			
(2) Social skill	0.86	0.543	0.327	1.000		
(3) Peer relationship	0.89	0.53	0.400	0.542	1.00	
(4) SWB	0.92	0.52	0.382	0.637	0.491	1.00

**Table 2:** Descriptive statistics and correlation analysis results.

Variables	Mean	SD	1	2	3	4
(1) Mindfulness	3.586	0.855	***0.719* ^+^**			
(2) Social skill	2.863	0.473	0.272 **	***0.737* ^+^**		
(3) Peer relationship	3.324	0.753	0.348 **	0.435 **	***0.725* ^+^**	
(4) SWB	3.637	0.675	0.346 **	0.571 **	0.432 **	***0.717* ^+^**

Note. N = 761; ** *p* < 0.01 (two tailed); ^+^ Values written in italics and bold are the square roots of the AVE values.

**Table 3:** Regression and mediation analysis results.

Dependent Variable (Social Skill)				
Variables	Coeff.	*p*	Bootstrap 95% CI	
Constant	2.535	0.00	2.154; 2.915	
Mindfulness	0.154	0.00	0.116; 0.192	
Age	−0.003	0.75	−0.026; 0.018	
Gender	−0.061	0.07	−0.128; 0.006	
Family Income	−0.043	0.13	−0.098; 0.013	
R^2^ = 0.081 *p* = 0.00				
**Dependent Variable (Peer relationships)**				
**Variables**	**Coeff.**	***p***	**Bootstrap 95% CI**	
Constant	0.969	0.00	0.364; 1.575	
Social skill	0.586	0.00	0.483; 0.688	
Mindfulness	0.219	0.00	0.162; 0.276	
Age	−0.008	0.59	−0.040; 0.023	
Gender	0.078	0.11	−0.019; 0.175	
Family Income	−0.042	0.29	−0.0123; 0.038	
R^2^ = 0.249 *p* = 0.00				
**Dependent Variable (SWB)**				
**Variables**	**Coeff.**	***p***	**Bootstrap 95% CI**	
Constant	0.906	0.00	0.462; 1.236	
Social skill	0.646	0.00	0.557; 0.736	
Peer relationships	0.159	0.00	0.101; 0.216	
Mindfulness	0.124	0.00	0.077; 0.172	
Age	0.023	0.074	−0.002; 0.048	
Gender	0.091	0.0126	−0.084; 0.169	
Family Income	−0.036	0.272	−0.100; 0.028	
R^2^ = 0.397 *p* = 0.00				
**Direct and Total Effects**				
**Effects**	**Paths**	**Effect**	***p***	**Bootstrap 95% CI**
Direct Effect	MF → SS	0.154	0.00	0.116; 0.192
Direct Effect	SS → PR	0.586	0.00	0.483; 0.688
Direct Effect	MF → PR	0.219	0.00	0.162; 0.276
Direct Effect	PR → SWB	0.159	0.00	0.101; 0.216
Direct Effect	SS → SWB	0.646	0.00	0.557; 0.736
Direct Effect	MF → SWB	0.124	0.00	0.077; 0.172
Total Effect	MF → SWB	0.273	0.00	0.220; 0.326
**Indirect Effects**		**Effect**	**Bootstrap 95% CI**	
Indirect Effect 1	MF → SS → SWB	0.099	0.064; 0.138	
Indirect Effect 2	MF → PR → SWB	0.035	0.019; 0.052	
Indirect Effect 3	MF → SS → PR → SWB	0.018	0.009; 0.029	
Total Indirect Effect		0.149	0.104; 0.195	

## Data Availability

The data presented in this study are available on request from the corresponding author due to ethical restrictions.

## Abbreviations

The following abbreviations are used in this manuscript:

SWB	Spiritual well-being
